# Content validity of guidance on self-care in the post-operative period for breast cancer

**DOI:** 10.1590/0034-7167-2024-0188

**Published:** 2024-09-20

**Authors:** Cristina Poliana Rolim Saraiva dos Santos, Natália Frota Goyanna, Erilaine de Freitas Corpes, Romel Jonathan Velasco Yanez, José Jeová Mourão, Régia Christina Moura Barbosa, Ana Fátima Carvalho Fernandes

**Affiliations:** I Universidade Federal do Ceará. Fortaleza, Ceará, Brazil; II Faculdade Luciano Feijão. Sobral, Ceará, Brazil

**Keywords:** Self Care, Postoperative Period, Validation Study, Breast Cancer, Women’s Health, Autocuidado, Periodo Posoperatorio, Estudio de Validación, Neoplasias de la Mama, Salud de la Mujer

## Abstract

**Objectives:**

to validate the content of a guidance guide on self-care in the postoperative period of breast surgery for breast cancer.

**Methods:**

a methodological study with content validity, carried out with 15 expert nurses and physiotherapists, between May and July 2022. Recruitment took place from the *Lattes* Platform, using snowball sampling. The level of relevance and representativeness for each item was verified using the Content Validity Index (CVI). It was considered valid when CVI was equal to or greater than 78% (0.78).

**Results:**

from the initial total of 37 items, two were excluded, as they had insufficient practical relevance and theoretical relevance, and another five items which, although presenting a CVI lower than the established cut-off, were suggested to be rewritten and grouped with other similar items.

**Conclusions:**

thirty items were considered valid and demonstrated important and significant characteristics, constituting suitable material for application in clinical practice.

## RESUMEN

**Objetivos:**

validar el contenido de una guía orientadora sobre autocuidado en el postoperatorio de cirugía mamaria por cáncer de mama.

**Métodos:**

estudio metodológico con validación de contenido, realizado con 15 enfermeros especialistas y fisioterapeutas, entre mayo y julio de 2022. El reclutamiento se realizó desde la Plataforma *Lattes*, mediante la técnica de bola de nieve. El nivel de relevancia y representatividad de cada ítem se verificó mediante el Índice de Validez de Contenido (IVC). Se consideró válido cuando el CVI era igual o superior al 78% (0,78).

**Resultados:**

del total inicial de 37 ítems, se excluyeron dos por no tener suficiente relevancia práctica y teórica, y otros cinco ítems que, si bien presentaban un CVI inferior al corte establecido, se sugirió reescribirlos y agruparlos con otros similares. elementos.

**Conclusiones:**

treinta ítems fueron considerados válidos y demostraron características importantes y significativas, constituyendo material apto para su aplicación en la práctica clínica.

## INTRODUCTION

Breast cancer is the second most common and fourth leading cause of cancer death in the world. Among women, it is the most commonly diagnosed type and the main cause of death from malignant neoplasia in the vast majority of countries. In 2022, there were 2.3 million new cases and around 670 thousand deaths from this cause. The estimate by the International Agency for Research on Cancer (IARC) indicates that the number of diagnoses will grow by 42.9% between 2022 and 2050. In Brazil, the forecast is for more than 146 thousand new cases in 2050, an increase of 54.4 % in relation to the almost 95 thousand cases estimated in 2022^(1)^.

Breast cancer treatment requires a multidisciplinary approach^(2)^, and is defined according to tumor location, age at presentation, staging, histopathological, biological, molecular and genetic criteria^(3)^. Therapeutic strategies include a combination of local approaches (surgery and radiotherapy) and systemic treatment (chemotherapy, hormone therapy, targeted drug therapy and immunotherapy). Surgery is the main treatment for early breast cancer and involves mastectomy or breast-conserving surgery^(4)^.

The complexity of treatment and the entire oncological context exacerbates women’s biopsychosocial-spiritual needs, requiring comprehensive interventions adapted to individual characteristics^(5)^. In this regard, these women need to be educated and empowered to exercise self-care, as this enhances the physical, social, psychological and spiritual dimensions of the quality of life of breast cancer patients^(6)^.

Considering the above, the relevance of this study is based on the importance of guiding women in the post-operative period of breast cancer, through the use of validated educational material regarding specific care for a smooth rehabilitation, contributing to knowledge and motivation in carrying out of self-care.

## OBJECTIVES

To construct and validate the content of a guidance guide on self-care in the postoperative period of breast surgery for breast cancer.

## METHODS

### Ethical aspects

The study was conducted in accordance with national (Resolution 466/12 of the Brazilian National Health Council) and international ethical guidelines, and was approved by the *Maternidade Assis Chateaubriand* Research Ethics Committee, *Universidade Federal do Ceará*, whose opinion is attached to this submission.

### Study design, period and location

This is a descriptive, methodological study, carried out from May to July 2022 at the *Maternidade Assis Chateaubriand, Universidade Federal do Ceará*, following the Standards for QUality Improvement Reporting Excellence (SQUIRE) guidelines.

This instrument is part of a larger research, specifically the doctoral thesis entitled “*Telenursing na promoção do autocuidado de mulheres no pós-operatório por câncer de mama: ensaio clínico randomizado*”.

### Sample, inclusion and exclusion criteria

The sample consisted of 15 healthcare professionals with academic and/or clinical experience in breast cancer and/or oncology and/or women’s health. Expert selection was carried out through the *Lattes* Platform on the Brazilian National Council for Scientific and Technological Development (CNPq - *Conselho Nacional de Desenvolvimento Científico e Tecnológico*) portal. Moreover, snowball sampling was carried out, in which experts were able to indicate other professionals^(7)^.

The requirements proposed by Jasper^(8)^ were used to assess the eligibility criteria to enter the study sample. Each criterion comprises different characteristics, namely: 1) skill/knowledge on the subject acquired through experience (professional care experience with oncology patients; teaching experience and participation in a research project in the area of interest); 2) specialized skill/knowledge(s), which made professionals an authority/expertise on the subject (having been a guest speaker at a national or international scientific event; supervisor of *Stricto Sensu* Graduate academic work(s); having master’s degree, with dissertation, and/or doctoral degree, with thesis on a topic related to the area of interest); 3) special ability in a certain study design (authorship of scientific article(s) with topics related to the area of interest in journal(s) classified by the Coordination for the Improvement of Higher Education Personnel (CAPES - *Coordenação de Aperfeiçoamento de Pessoal de Nível Superior*); participation in assessment committee(s) of *Stricto Sensu* Graduate academic work(s) with topics related to the area of interest; 4) passing a specific test to identify judges (being a professional recognized by the Federal Nursing Council as an oncologist nurse or holding a specific exam with proof of long experience in oncology); 5) high classification attributed by an authority (having received, from a well-known scientific institution, an honorable mention of recognition as an authority in the area of interest; having work(s) awarded in a national or international event(s) whose contents refer to the area of interest).

It was stipulated that each expert judge met at least two criteria. Participants who met the eligibility criteria were sent an invitation letter by email, with information about the purpose of the research and the methods adopted, inviting them to participate in the study. Upon acceptance, the Informed Consent Form (ICF) was obtained from all individuals involved in the study online, through contact via email, and they were instructed on how to return it to the researcher after signing for subsequent sending of the material to be examined.

### Guidance guide construction method

#### First stage: item construction

The first stage consisted of developing an initial instrument with 37 items with post-operative self-care guidelines aimed at treating breast cancer. Initially, a literature review was carried out, and, based on information found in scientific articles and manuals^(9-25)^, a script was developed with post-operative self-care actions aimed at treating breast cancer. The items in this script cover the topics of surgical wound, suction drain, arm ipsilateral to surgery, prevention of lymphedema, nutrition, hygiene, physical, social, psychological rehabilitation and use of medications, and will constitute educational material aimed at patients, being presented in the form of an orientation guide.

#### Second stage: guidance guide validity

Three materials were sent to expert judges. The first was entitled “Characterization of content expert judges”, with questions related to the identification of individual sociodemographic variables, such as name, gender, age, city where they work, and academic/professional variables, which included place and year of graduation, academic title (undergraduate, graduate education, experience with the topic breast cancer, participation in research projects and professional trajectory (institution and length of experience)). The second was called “Instructions for judges regarding content validity”, with information on the criteria and scores for assessing the instrument items. And the third was entitled “Instrument content assessment regarding language clarity, practical relevance and theoretical relevance”, with items on post-operative self-care of breast cancer surgeries to be assessed.

A total of 37 items from the initial script were assessed, whose domains are related to surgical wound care (items 1 to 7; 27 to 29), suction drain (items 8 to 12; 30), ipsilateral arm/lymphedema prevention (items 13 to 19), food (items 20 to 23; 3 and 33), physical, social, psychosocial rehabilitation (items 24 to 26; 31) and medications (items 34 to 37).

Once this stage was completed by experts, modifications to the items followed the suggestions regarding changes to some words with appropriate synonyms, position of words in the sentence, punctuation, order of information, addition of words to better explain the item, but there were also suggestions for clustering similar items, and thus, the final configuration of the instrument consisted of 30 items, which remained distributed among the initially proposed domains.

Once total data collection was completed, judges were characterized and the items in the guidance guide were adapted. The data obtained was organized, processed and analyzed using Microsoft Excel^®^ 2016. Descriptive statistical analysis was performed using absolute and relative frequency.

#### Analysis of results, and statistics

Similar to the assessment carried out in the study by Matos *et al*.^(26)^, a Likert scale was used to adequately score the instrument questions according to language clarity (LC), practical relevance (PR) and theoretical relevance (TR) categories, and the answers followed the degree of agreement with the criteria so that 1 represented “very little relevant”, 2 represented “little relevant”, 3 represented “moderately relevant”, 4 represented “relevant” and 5 represented “very relevant”. Furthermore, the instrument presented a column for additional suggestions from evaluators. In this case, it was considered relevant when the scores were 4 or 5 on a five-point ordinal scale.

Each of the instrument’s 37 items was assessed according to the three dimensions: LC; PR; and TR. The level of relevance and representativeness for each of the instrument’s items was verified, using the Content Validity Index (CVI), considered valid when the approval rate was equal to or greater than 78% (0.78), value that guided decisions about revisions or rejections of items^(27-28)^. It was decided that a CVI lower than this cut-off point, in its three dimensions, would mean immediate exclusion of an item, with no chance of adjustments. However, when this value occurred in up to two criteria, an item should be adjusted as indicated by expert judges.

## RESULTS

Initially, 42 invitations were sent to evaluators from different professional categories. Of these, 23 did not return the signed ICF, but 19 returned the email with the signed document, confirming their participation; however, despite accepting the invitation, four did not send the assessed instrument within the stipulated deadlines. In the end, 15 expert judges analyzed the instrument.

Of the judges who made up the final sample, 80% were nurses and 20% were physiotherapists. The sample was characterized by participants aged between 22 and 53 years old, with a median of 34 years old (SD± 8.8). Regarding professional training time, participants had a median of 9.5 years (SD±9.8). The majority were female (93.3%) and from the Northeast (93.3%). Regarding degrees, 53.3% held masters’ or doctoral degrees and 33.3% were expert nurses or had a residency in cancerology/oncology. It should be noted that 20% held a master’s degree and 13.3% held a doctoral degree.

As for current professional occupation, it was found that 26.7% of the sample worked in care and research; 20% worked in teaching and research; and 6.6% worked in care, teaching and research. In care alone, 26.7% were quantified, and in research alone, 20%. All had experience with the topic of breast cancer, with a median of 5.77 years (SD± 6.9). It should be added that 40% of expert judges reported experience in validating materials in the health area. Concerning scientific production, 66.7% of judges had research or publications in the area of interest (women’s health, oncology, breast cancer, telenursing).

Table 1 shows the characterization data of content expert judges listed for this study according to Jasper^(8)^ criteria. It should be noted that an expert judge may have accumulated more than one classification criterion.

**Table 1:** Characterization of content expert judges participating in the study according to the criteria proposed by Jasper^(7)^, Fortaleza, Ceará, Brazil, 2022

Judges’ classification criteria (N=15)	n	%
Has skills/knowledge acquired through experience	15	100.0
Has specialized skill/knowledge(s) that make professionals an authority on the subject	07	46.7
Has special ability in a certain type of study	11	73.3
Passed a specific test to identify judges	04	26.7
Has a high rating from an authority	-	-

In relation to Jasper’s criteria^(8)^, 15 judges (100%) who participated in validating the guidelines met the minimum requirements, with two judges (13.3%) meeting eight characteristics, another three judges (20%), five characteristics, three more judges (20%), four characteristics, five judges (33.3%), three characteristics and, finally, two judges (13.3%), two characteristics. Thus, according to the distribution of judges’ scores, the two most prevalent criteria were skill/knowledge acquired through experience (100%) and special skill in a certain study design (73.3%).

The guide with guidance on post-operative self-care after breast cancer surgery, initially constructed with 37 items, after content validity by expert judges, underwent adjustments. Items 31 and 36 were extracted, as they obtained a CVI value <0.78 (established cut-off point value) in their three dimensions (LC, PR, TR). However, for items that did not obtain an appropriate CVI value for this study, in any of the three dimensions, they were modified according to suggestions from judges and relevant literature.

The LC dimension presented the largest number of items with insufficient CVI value (2, 6, 7, 10, 13, 14, 17, 28, 31, 34, 35, 36, 37), i.e., some expert judges disagreed regarding to the language used to assess the item negatively, which implied the need to adapt it according to respondents’ suggestions. For PR, except for the removed items, no other item obtained CVI < 0.78, meaning that, in this judgment, each item was considered important to be included in the assessed instrument. As for TR, only items 20 and 23 presented CVI < 0.78, demonstrating that their theoretical contents are not representative according to experts’ assessment.

The data expressed in Table 2 show judges’ judgment regarding the LC, PR and TR dimensions, according to the CVI.

**Table 2:** Content Validity Index for clarity of language, practical relevance and theoretical relevance of instrument items applied to expert judges, Fortaleza, Ceará, Brazil, 2022

Items	LC	PR	TR
*Lave as mãos com água e sabão ao manusear a ferida operatória.*	0.80	1.00	1.00
*Inspecione a ferida para detectar possíveis sinais de infecção (vermelhidão, calor, secreção purulenta, febre).*	0.67^*^	1.00	1.00
*Descubra a ferida após 24 horas da cirurgia.*	0.87	1.00	1.00
*Evite a força de jato de água na pele e pressão sobre o local.*	0.87	1.00	1.00
*Mantenha a ferida sempre limpa e seca evitando quaisquer produtos (óleos, pomadas, spray), a menos que orientada pela equipe de saúde.*	0.93	1.00	1.00
*Evite uso de fitas adesivas direto na pele perincisional.*	0.67^*^	1.00	1.00
*Evite exposição solar.*	0.73^*^	0.87	0.87
*Realize esvaziamento e verifique o volume da secreção e anote em formulário próprio fornecido na alta hospitalar.*	0.80	0.93	0.93
*Mantenha o dispositivo abaixo do local da inserção cirúrgica do mesmo, sem que fique direto no chão, pendurado ou tracionado.*	0.87	0.93	0.87
*Realize a limpeza ao redor do local de inserção após o banho.*	0.64^*^	0.93	0.93
*Use roupas largas e abertas na frente.*	0.93	0.93	0.93
*Seque a pele ao redor do local de inserção do dreno após o banho.*	0.86	0.93	0.93
*Evite a exposição ao calor, machucados e queimaduras, aferição de pressão arterial e/ou glicemia capilar e/ou injeções e/ou retirada de sangue.*	0.53^*^	0.80	0.87
*Evite retirada de cutículas.*	0.73^*^	0.80	0.87
*Evite dormir por cima do lado operado.*	0.93	0.93	0.93
*Use luvas ao mexer em plantas ou no forno.*	0.87	0.80	0.80
*Use dedal ao costurar e meias elásticas no braço do lado operado.*	0.73^*^	0.80	0.80
*Evite sobrecarga e a não realize movimentos bruscos, de longa duração e repetitivo.*	0.80	0.93	0.87
*Inicie fisioterapia.*	0.87	1.00	1.00
*Reduza o consumo de gordura de origem animal na alimentação.*	0.80	0.80	0.73^*^
*Aumente o consumo de frutas, vegetais, hortaliças.*	0.87	0.93	0.87
*Consuma bastante líquido.*	0.80	1.00	1.00
*Evite massas e doces.*	0.80	0.80	0.73^*^
*Busque rede de apoio com profissionais habilitados (psicólogo, fisioterapeuta, nutricionista, assistente social, enfermeiro, por exemplo).*	0.93	1.00	1.00
*Converse com familiares sobre medos, anseios, expectativas, sentimentos, preocupações.*	1.00	0.93	0.87
*Realize atividade física leve como pequenas caminhadas*	0.80	0.93	0.93
*Tome banho diariamente e use sabonetes neutros na região operada (ph 5,4 a 5,6).*	0.80	0.87	0.87
*Mantenha a pele limpa e hidratada.*	0.60^*^	0.80	0.80
*Troque a roupa diariamente.*	0.87	0.78	0.86
*Durma na posição dorsal (barriga pra cima) evitando acotovelamento do dreno e pressão sobre a ferida operatória.*	0.93	1.00	1.00
*Deixe o ambiente silencioso e tranquilo.*	0.61^*^	0.61^*^	0.61^*^
*Realize pequenas refeições frequentes para reduzir sensação de estômago cheio.*	0.85	0.92	1.00
*Evite deitar logo após uma alimentação*	0.92	0.85	0.85
*Faça uso da medicação caso tenha sido prescrita pelo médico.*	0.71^*^	0.93	0.93
*Identifique efeitos colaterais, posologia, horários.*	0.57^*^	0.78	0.86
*Leia a bula dos medicamentos prescritos.*	0.46^*^	0.53^*^	0.53^*^
*Observe aparecimento de sinais de alergia após uso dessas medicações e comunique a equipe.* CVI of items without CVI <0.78 Overall CVI without CVI <0.78	0.77^*^ 0.86 0.90	0.85 0.90	0.85 0.91

Notes: LC - language clarity; PR - practical relevance; TR - theoretical relevance; CVI - Content Validity Index; (^*^) CVI lower than the cutoff point (0.78).

Once this stage of assessment by experts was completed, modifications to guide items followed the suggestions regarding changes to some words with appropriate synonyms, position of words in the sentence, punctuation, order of information, addition of words to better explain the item, but there were also suggestions of grouping similar items together. Thus, the final configuration consisted of 30 items (Chart 1).

**Chart 1:** Instrument items with guidance on post-operative self-care after breast cancer surgery after modification, as suggested by experts, Fortaleza, Ceará, Brazil, 2022

*Lave as mãos com água e sabão antes de tocar na ferida operatória.*
*Observe a ferida operatória para identificar possíveis sinais de infecção como vermelhidão, calor, secreção amarelada ou amarelo-esverdeada, febre.*
*Retire o curativo e deixe a ferida operatória descoberta após 24 horas da cirurgia.*
*Evite jato forte de água na pele e qualquer outro tipo de pressão sobre o local.*
*Mantenha a ferida operatória sempre limpa e seca evitando quaisquer produtos (óleos, pomadas, spray) a menos que orientada pela equipe de saúde.*
*Evite uso de fitas adesivas (esparadrapo, micropore) direto na pele ao redor do local da cirurgia.*
*Evite pegar sol na região da cirurgia.* *Tome banho todo dia e use sabonete líquido neutro (ph 5,4 a 5,6) na região operada.*
*Realize esvaziamento do dreno, medindo o volume da secreção retirada com uma seringa e anote no formulário fornecido na alta hospitalar. Logo após, despreze a secreção no sanitário.*
*Mantenha o dreno abaixo do local da cirurgia, evitando que fique direto no chão, pendurado ou dobrado.*
*Use roupas confortáveis, largas e abertas na frente para acomodar melhor o posicionamento do dreno. Troque-as diariamente.*
*Seque a pele ao redor do local onde o dreno está inserido, após o banho, com uma toalha limpa.*
*Durma com a barriga para cima evitando dobrar, acotovelar ou pressionar o dreno sobre a ferida operatória.*
*Evite exposição do braço do lado operado ao calor, machucados, queimaduras, aferição de pressão arterial e/ou glicemia capilar e/ou injeções e/ou retirada de sangue.*
*Evite retirada de cutículas das unhas das mãos do lado operado.*
*Use luvas de proteção térmica ao mexer no forno e luvas grossas ao mexer em plantas.*
*Use dedal ao costurar e meias elásticas no braço do lado operado conforme orientação profissional.*
*Evite pegar peso e não realize movimentos bruscos, de longa duração e repetitivo no braço do lado operado.*
*Diminua o consumo de gordura de origem animal na alimentação e mantenha as orientações nutricionais.*
*Dê preferência ao consumo de frutas, vegetais, hortaliças e mantenha as orientações nutricionais.*
*Beba bastante água, sucos naturais de frutas, evitando refrigerantes, bebidas alcoólicas e sucos artificiais.*
*Evite massas e doces e mantenha as orientações nutricionais.*
*Realize pequenas refeições ao longo do dia para reduzir sensação de estômago cheio.*
*Evite deitar logo após uma alimentação.*
*Busque rede de apoio com profissionais habilitados (psicólogo, fisioterapeuta, nutricionista, assistente social, enfermeiro, por exemplo).*
*Converse com familiares sobre medos, anseios, expectativas, sentimentos, preocupações.*
*Inicie fisioterapia o mais precoce possível e após liberação médica.*
*Realize atividade física leve como pequenas caminhadas, pilates, hidroginástica, ioga, preferencialmente após orientação de profissional habilitado.*
*Tome a medicação prescrita pelo médico.*
*Identifique possíveis efeitos colaterais e sinais de alergia das medicações tomadas, informando à equipe de saúde caso ocorra.*

## DISCUSSION

The educational guide validity previously presented demonstrated important and significant characteristics, being considered adequate, whereas, in most items, only an adjustment in language was suggested, which refers exclusively to the way the sentence is written with a focus on objectivity and clarity, without using ambiguous terms, which may represent inadequate and imprecise understandings^(29)^.

In the other two domains, PR and TR, minor considerations were highlighted by experts, leading to two conclusions: the first concerns the practical importance of the issues explored in the material, taking into account that, knowing the particularities of the target population and the current problem health, there is empowerment on the subject addressed; and the second refers to the essentiality of the items remaining in the instrument, as they fulfill the efficient educational purpose proposed.

Another observation refers to the way content validity is measured, as, being carried out by CVI, it allows analyzing each domain, component, item and the instrument as a whole, considering this a measure widely disseminated and accepted in the literature^(30)^.

The inclusion of another healthcare professional category as an expert was based on the principles of interdisciplinarity and multidisciplinarity, in addition to this diversity qualifying the methodological path of construct validity, considering that this decision contributed to the mitigation of imprecise results and erroneous conclusions or biased measures^(29)^.

Even so, nurses made up a larger portion of evaluators, certifying nursing as a science under construction due to its character of creation and change by combining scientific knowledge with technical procedures, and this constant search for expanding the spheres of care brings positive perspectives to the profession, based on the intentionality of developing products capable of promoting, maintaining and rehabilitating health in different scenarios.

Considering the educational process, the recognition of individuals must be perceived as a necessary factor in health practices, reorienting the current model and making them a participant in the care proposal^(31)^.

In the breast oncology scenario, from screening and early detection, extending throughout the treatment process, information transfer must occur in a simple way, as appropriate health behaviors can be achieved by encouraging self-care so that, in this way, there is a possibility of individual autonomy^(32)^.

Thus, cancer patients need care in which their health condition is monitored, controlled and can be continuously monitored within an educational process in search of self-care and transformation of individual behavior to promote health, and this measure is capable of to produce significant changes in the ability to adjust to the new health condition, including personal empowerment^(33)^.

It is understood that the levels of self-care and management of cancer patients deserve to be improved through nursing planning, in order to improve prognoses from a physical point of view and increase their psychological flexibility to form a bidirectional benign complementary structure of physiology and psychology, since rehabilitation includes the entirety of human beings^(34)^. In this way, many tools can be used to achieve education and health promotion, but, for this, educational instruments need to be updated, expanded, improved and constantly assessed, considering the weaknesses of the moment.

### Study limitations

This study only validated the content of a guidance guide, other forms of validity being excluded. Another limitation is the fact that expert judges are from just two professional categories, nursing and physiotherapy, since the participation of other categories could bring other perspectives, qualifying and expanding the scope of the educational material.

### Contributions to nursing, health, or public policy

Potential contributions to the field of health practices are based on the possibility of this guidance guide offering support to professionals for education of post-operative breast cancer patients, contributing to a better recovery and rehabilitation of these women. Furthermore, its use provides dissemination of knowledge and can be a strategy that encourages self-care.

## CONCLUSIONS

The content of a guidance guide on post-operative self-care in breast cancer treatment was considered adequate and valid by experts. The suggestions made by the evaluators were included and refined, being suitable for application in clinical practice with the population of interest.
