# Supplementary material for: Content validity of guidance on self-care in the post-operative period for breast cancer
Source: Rev Bras Enferm. 2024 Sep 20;77(4):e20240188. doi: 10.1590/0034-7167-2024-0188 (PMC11419679; doi:10.1590/0034-7167-2024-0188)
Supplement: Translation

# Translation: Validação de conteúdo de guia de orientação sobre autocuidado no pós-operatório de câncer de mama

**DOI:** 10.1590/0034-7167-2024-0188pt

**Authors:** Cristina Poliana Rolim Saraiva dos Santos, Natália Frota Goyanna, Erilaine de Freitas Corpes, Romel Jonathan Velasco Yanez, José Jeová Mourão, Régia Christina Moura Barbosa, Ana Fátima Carvalho Fernandes

**Affiliations:** I Universidade Federal do Ceará. Fortaleza, Ceará, Brasil; II Faculdade Luciano Feijão. Sobral, Ceará, Brasil

**Keywords:** Autocuidado, Período Pós-Operatório, Estudo de Validação, Câncer de Mama, Saúde da Mulher

## Abstract

**Objetivos:**

validar o conteúdo de um guia de orientação sobre autocuidado no pós-operatório de cirurgia mamária por câncer de mama.

**Métodos:**

estudo metodológico com validação de conteúdo, realizado com 15 enfermeiros e fisioterapeutas especialistas, entre maio e julho de 2022. O recrutamento ocorreu a partir da Plataforma *Lattes*, por meio da técnica *snowball*. Verificou-se o nível de relevância e representatividade para cada item por meio do Índice de Validade de Conteúdo (IVC). Considerou-se válido quando o IVC foi igual ou superior a 78% (0,78).

**Resultados:**

do total inicial de 37 itens, foram excluídos dois, por apresentarem pertinência prática e relevância teórica insuficientes, e outros cinco itens que, embora apresentando IVC inferior ao corte estabelecido, seguiu-se a sugestão da reescrita e agrupamento a outros itens semelhantes.

**Conclusões:**

trinta itens foram considerados válidos e demonstraram características importantes e significativas, configurando-se material adequado para aplicação na prática clínica.

## INTRODUÇÃO

O câncer de mama é o segundo mais incidente e a quarta principal causa de morte por câncer no mundo. Entre as mulheres, é o tipo mais comumente diagnosticado e a principal causa de óbito por neoplasia maligna na grande maioria dos países. Em 2022, ocorreram 2,3 milhões de casos novos e cerca de 670 mil óbitos por essa causa. A estimativa da *International Agency for Research on Cancer* (IARC) aponta que o número de diagnósticos crescerá 42,9% entre 2022 e 2050. No Brasil, a previsão é de mais de 146 mil novos casos em 2050, um aumento de 54,4% em relação aos quase 95 mil casos estimados em 2022^(1)^.

O tratamento do câncer de mama requer uma abordagem multiprofissional^(2)^, e é definido de acordo com a localização do tumor, idade de apresentação, estadiamento, critérios histopatológicos, biológicos, moleculares e genéticos^(3)^. As estratégias terapêuticas incluem uma combinação de abordagens locais (cirurgia e radioterapia) e tratamento sistêmico (quimioterapia, hormonioterapia, terapia medicamentosa direcionada e imunoterapia). A cirurgia é o principal tratamento do câncer de mama inicial e envolve mastectomia ou cirurgia conservadora da mama^(4)^.

A complexidade do tratamento e todo o contexto oncológico exacerba necessidades biopsicossocioespirituais das mulheres, sendo necessárias intervenções abrangentes e adaptadas às características individuais^(5)^. Nesse sentido, essas mulheres necessitam ser educadas e empoderadas para o exercício do autocuidado, uma vez que isso potencializa as dimensões físicas, sociais, psicológicas e espirituais da qualidade de vida das pacientes com câncer de mama^(6)^.

Diante do exposto, a relevância deste estudo se assenta na importância de orientar mulheres no pós-operatório de câncer de mama, por meio da utilização de um material educativo validado quanto a cuidados específicos para uma reabilitação tranquila, contribuindo para o conhecimento e motivação na realização do autocuidado.

## OBJETIVOS

Construir e validar o conteúdo de um guia de orientação sobre autocuidado no pós-operatório de cirurgia mamária por câncer de mama.

## MÉTODOS

### Desenho, período e local do estudo

Trata-se de estudo descritivo, do tipo metodológico, realizado no período de maio a julho de 2022 na Maternidade Assis Chateaubriand, da Universidade Federal do Ceará, seguindo as diretrizes do *Standards for QUality Improvement Reporting Excellence* (SQUIRE).

O referido instrumento configura-se parte de uma pesquisa maior, especificamente a tese de doutoramento intitulada “*Telenursing* na promoção do autocuidado de mulheres no pós-operatório por câncer de mama: ensaio clínico randomizado”.

### Amostra, critérios de inclusão e exclusão

A amostra foi composta por 15 profissionais de saúde com experiência acadêmica e/ou clínica em câncer de mama e/ou oncologia e/ou saúde da mulher. A seleção dos especialistas foi realizada por meio da Plataforma *Lattes* do portal do Conselho Nacional de Desenvolvimento Científico e Tecnológico (CNPq). Além disso, foi realizada a amostragem *snowball*, na qual os especialistas puderam indicar outros profissionais^(7)^.

Os requisitos propostos por Jasper^(8)^ foram utilizados a fim de avaliar os critérios de elegibilidade para entrar na amostra do estudo. Cada critério compreende diferentes características, a saber: 1) habilidade/conhecimento na temática adquirido pela experiência (experiência profissional assistencial junto a pacientes oncológicos; experiência docente e participação de projeto de pesquisa na área de interesse); 2) habilidade/conhecimento especializado(s), que tornaram o profissional uma autoridade/*expertise* no assunto (ter sido palestrante convidado em evento científico nacional ou internacional; orientador de trabalho(s) acadêmico(s) de Pós-Graduação *Stricto Sensu*; ter título de mestre, com dissertação, e/ou título de doutor, com tese em temática relativa à área de interesse); 3) habilidade especial em determinado tipo de estudo (ter autoria de artigo(s) científico(s) com temáticas relativas à área de interesse em periódico(s) classificados pela Coordenação de Aperfeiçoamento de Pessoal de Nível Superior (CAPES); participação em banca(s) avaliadora(s) de trabalho(s) acadêmico(s) de Pós-Graduação *Stricto Sensu* com temáticas relativas à área de interesse); 4) aprovação em um teste específico para identificar juízes (ser profissional reconhecido pelo Conselho Federal de Enfermagem como enfermeiro oncologista ou titulado em exame específico mediante comprovação de longa atuação na oncologia); 5) classificação alta atribuída por uma autoridade (ter recebido de instituição científica conhecida, homenagem menção honrosa de reconhecimento como autoridade na área de interesse; possuir trabalho(s) premiado(s) em evento(s) nacional(is) ou internacional(is) cujos conteúdos sejam referentes à área de interesse).

Foi estipulado que cada juiz especialista atendesse a, pelo menos, dois critérios. Aos participantes que preencheram os critérios de elegibilidade, foi enviada uma carta convite, por correio eletrônico, com informações sobre a finalidade da pesquisa e os métodos adotados, convidando-os a participar do estudo. Após o aceite, o Termo de Consentimento Livre e Esclarecido (TCLE) foi obtido de todos os indivíduos envolvidos no estudo de forma *online*, por meio de contato via e-mail, sendo orientados sobre a devolução à pesquisadora após assinatura para posterior envio do material a ser examinado.

### Método de construção do guia de orientação

#### Primeira etapa: construção dos itens

A primeira etapa consistiu na elaboração de instrumento inicial com 37 itens com orientações de autocuidado pós-operatório direcionado ao tratamento do câncer de mama. Inicialmente, foi realizada revisão de literatura, e, a partir de informações encontradas em artigos científicos e manuais^(9-25)^, foi desenvolvido roteiro com ações de autocuidado pós-operatório direcionado ao tratamento do câncer de mama. Os itens desse roteiro contemplam os temas sobre ferida operatória, dreno de sucção, braço homolateral à cirurgia, prevenção de linfedema, alimentação, higiene, reabilitação física, social, psicológica e uso de medicamentos, e constituirão um material educativo voltado para pacientes, sendo apresentado na forma de um guia de orientação.

#### Segunda etapa: validação do guia de orientação

Três materiais foram encaminhados para os juízes especialistas. O primeiro intitulou-se “Caracterização dos juízes especialistas de conteúdo”, com perguntas relacionadas à identificação de variáveis sociodemográficas individuais, como nome, gênero, idade, cidade onde trabalha, e variáveis acadêmicas/profissionais, que incluíram local e ano de conclusão da graduação, titulação acadêmica (graduação, pós-graduação, experiência com o tema câncer de mama, participação em projetos de pesquisa e trajetória profissional (instituição e tempo de atuação)). O segundo denominou-se “Instruções para os juízes quanto à validação de conteúdo”, com informações sobre os critérios e pontuação para avaliação dos itens do instrumento. E o terceiro intitulou-se “Avaliação de conteúdo de instrumento quanto clareza de linguagem, pertinência prática e relevância teórica”, com os itens sobre autocuidado pós-operatório de cirurgias por câncer de mama a serem avaliados.

Foram avaliados 37 itens do roteiro inicial, cujos domínios são relativos aos cuidados com ferida operatória (itens 1 a 7; 27 a 29), dreno de sucção (itens 8 a 12; 30), braço homolateral/prevenção de linfedema (itens 13 a 19), alimentação (itens 20 a 23; 3 e 33), reabilitação física, social, psicossocial (itens 24 a 26; 31) e medicamentos (itens 34 a 37).

Concluída essa etapa pelos especialistas, modificações nos itens seguiram as sugestões no tocante a alterações de algumas palavras por sinônimos apropriados, posição das palavras na frase, pontuação, ordem das informações, acréscimo de palavras para melhor explicação do item, mas também houve sugestões de agrupamento de itens semelhantes, e, assim, a configuração final do instrumento constou de 30 itens, que permaneceram distribuídos entre os domínios inicialmente propostos.

Finalizada a coleta total de dados, procedeu-se à caracterização dos juízes e adequação dos itens do guia de orientação. Os dados obtidos foram organizados, processados e analisados a partir do *Microsoft Excel^®^* 2016. A análise estatística descritiva foi realizada usando frequência absoluta e relativa.

#### Análise dos resultados e estatística

Semelhante à avaliação realizada no estudo de Matos *et al*.^(26)^, uma escala Likert foi utilizada para pontuar adequadamente os quesitos do instrumento conforme as categorias de clareza da linguagem (CL), pertinência prática (PP) e relevância teórica (RT), e as respostas seguiram o grau de concordância aos critérios, de forma que 1 representou “pouquíssimo relevante”, 2 representou “pouco relevante”, 3 representou “moderadamente relevante”, 4 representou “muito relevante” e 5 representou “muitíssimo relevante”. Além disso, o instrumento apresentou uma coluna destinada às sugestões adicionais dos avaliadores. Nesse caso, considerou-se relevante quando os escores foram de 4 ou 5 em uma escala ordinal de cinco pontos.

Cada um dos 37 itens do instrumento foi avaliado conforme as três dimensões: CL; PP; e RT. Verificou-se o nível de relevância e representatividade para cada um dos itens do instrumento, por meio do Índice de Validade de Conteúdo (IVC), considerando-se válido quando o índice de aprovação foi igual ou superior a 78% (0,78), valor que norteou as decisões sobre revisões ou rejeições de itens^(27-28)^. Decidiu-se que IVC inferior a esse ponto de corte, em suas três dimensões, significaria imediata exclusão do item, sem chances de ajustes, entretanto, quando esse valor ocorresse em até dois critérios, o item deveria ser ajustado conforme indicação dos juízes especialistas.

## RESULTADOS

Inicialmente, foram enviados 42 convites para avaliadores de diferentes categorias profissionais. Desses, 23 não devolveram o TCLE assinado, mas 19 retornaram o e-mail com o documento assinado, confirmando sua participação; entretanto, apesar de terem aceitado o convite, quatro não enviaram o instrumento avaliado dentro dos prazos estipulados. Ao final, 15 juízes especialistas analisaram o instrumento.

Dos juízes que compuseram a amostra final, 80% eram enfermeiros e 20% eram fisioterapeutas. A amostra caracterizou-se por participantes com idade entre 22 e 53 anos, sendo a mediana de 34 anos (DP± 8,8). Referente ao tempo de formação profissional, os participantes tinham mediana de 9,5 anos (DP±9,8). A maioria era do sexo feminino (93,3%) e procedente da Região Nordeste (93,3%). Quanto à titulação, 53,3% eram mestres ou doutores e 33,3% eram enfermeiras especialistas ou tinham residência em cancerologia/oncologia. Ressalta-se que 20% estavam cursando o mestrado e 13,3% estavam cursando o doutorado.

Em relação à ocupação profissional atual, verificou-se que 26,7% da amostra trabalhava na assistência e pesquisa; 20% trabalhava no ensino e pesquisa; e 6,6% trabalhava na assistência, ensino e pesquisa. Apenas na assistência, quantificaram-se 26,7%, e apenas na pesquisa, 20%. Todos possuíam experiência com a temática câncer de mama, com mediana de 5,77 anos (DP± 6,9). Acrescenta-se que 40% dos juízes especialistas referiram experiência em validação de materiais na área da saúde. Concernente à produção científica, 66,7% dos juízes possuíam pesquisas ou publicações na área de interesse (saúde da mulher, oncologia, câncer de mama, telenfermagem).

Observam-se, na Tabela 1, os dados da caracterização dos juízes especialistas de conteúdo elencados para este estudo segundo os critérios de Jasper^(8)^. Ressalta-se que um juiz especialista pode ter acumulado mais de um critério de classificação.

**Tabela 1:** Caracterização dos juízes especialistas de conteúdo participantes do estudo segundo os critérios propostos por Jasper^(7)^, Fortaleza, Ceará, Brasil, 2022

Critérios de classificação dos juízes (N=15)	n	%
Possuir habilidade/conhecimento adquirido(s) pela experiência	15	100,0
Possuir habilidade/conhecimento especializado(s) que tornam o profissional uma autoridade no assunto	07	46,7
Possuir habilidade especial em determinado tipo de estudo	11	73,3
Possuir aprovação em um teste específico para identificar juízes	04	26,7
Possuir classificação alta atribuída por uma autoridade	-	-

Com relação aos critérios de Jasper^(8)^, 15 juízes (100%) que participaram da validação das orientações atingiram os requisitos mínimos exigidos, sendo que dois juízes (13,3%) se enquadraram em oito características, outros três juízes (20%), em cinco características, mais três juízes (20%), em quatro características, cinco juízes (33,3%), em três características e, por fim, dois juízes (13,3%), em duas características. Dessa forma, de acordo com a distribuição da pontuação dos juízes, os dois critérios mais prevalentes foram habilidade/conhecimento adquirido(s) pela experiência (100%) e habilidade especial em determinado tipo de estudo (73,3%).

O guia com orientações sobre autocuidado pós-operatório de cirurgia por câncer de mama, inicialmente construído com 37 itens, após a validação de conteúdo por juízes especialistas, passou por ajustes. Foram extraídos os itens 31 e 36, já que obtiveram valor de IVC <0,78 (valor do ponto de corte estabelecido) em suas três dimensões (CL, PP, RT). Entretanto, para itens que não obtiveram valor de IVC apropriado para este estudo, em alguma das três dimensões, foram modificados conforme sugestões dos juízes e literatura pertinente.

A dimensão CL apresentou o maior número de itens com valor insuficiente do IVC (2, 6, 7, 10, 13, 14, 17, 28, 31, 34, 35, 36, 37), ou seja, alguns juízes especialistas discordaram quanto à linguagem utilizada avaliando o item negativamente, o que implicou necessidade de adequação do mesmo conforme sugestões dos respondentes. Para a PP, excetuando-se os itens removidos, nenhum outro obteve IVC < 0,78 significando que, nesse julgamento, cada item foi considerado como importante para constar no instrumento avaliado. Já para a RT, apenas os itens 20 e 23 apresentaram IVC < 0,78, demonstrando que os conteúdos teóricos destes não são representativos segundo avaliação dos especialistas.

Os dados expressos na Tabela 2 evidenciam o julgamento dos juízes quanto às dimensões CL, PP e RT, conforme o IVC.

**Tabela 2:** Índice de Validade de Conteúdo para clareza de linguagem, pertinência prática e relevância teórica dos itens do instrumento aplicado aos juízes especialistas, Fortaleza, Ceará, Brasil, 2022

Itens	CL	PP	RT
Lave as mãos com água e sabão ao manusear a ferida operatória.	0,80	1,00	1,00
Inspecione a ferida para detectar possíveis sinais de infecção (vermelhidão, calor, secreção purulenta, febre).	0,67^*^	1,00	1,00
Descubra a ferida após 24 horas da cirurgia.	0,87	1,00	1,00
Evite a força de jato de água na pele e pressão sobre o local.	0,87	1,00	1,00
Mantenha a ferida sempre limpa e seca evitando quaisquer produtos (óleos, pomadas, spray), a menos que orientada pela equipe de saúde.	0,93	1,00	1,00
Evite uso de fitas adesivas direto na pele perincisional.	0,67^*^	1,00	1,00
Evite exposição solar.	0,73^*^	0,87	0,87
Realize esvaziamento e verifique o volume da secreção e anote em formulário próprio fornecido na alta hospitalar.	0,80	0,93	0,93
Mantenha o dispositivo abaixo do local da inserção cirúrgica do mesmo, sem que fique direto no chão, pendurado ou tracionado.	0,87	0,93	0,87
Realize a limpeza ao redor do local de inserção após o banho.	0,64^*^	0,93	0,93
Use roupas largas e abertas na frente.	0,93	0,93	0,93
Seque a pele ao redor do local de inserção do dreno após o banho.	0,86	0,93	0,93
Evite a exposição ao calor, machucados e queimaduras, aferição de pressão arterial e/ou glicemia capilar e/ou injeções e/ou retirada de sangue.	0,53^*^	0,80	0,87
Evite retirada de cutículas.	0,73^*^	0,80	0,87
Evite dormir por cima do lado operado.	0,93	0,93	0,93
Use luvas ao mexer em plantas ou no forno.	0,87	0,80	0,80
Use dedal ao costurar e meias elásticas no braço do lado operado.	0,73^*^	0,80	0,80
Evite sobrecarga e a não realize movimentos bruscos, de longa duração e repetitivo.	0,80	0,93	0,87
Inicie fisioterapia.	0,87	1,00	1,00
Reduza o consumo de gordura de origem animal na alimentação.	0,80	0,80	0,73^*^
Aumente o consumo de frutas, vegetais, hortaliças.	0,87	0,93	0,87
Consuma bastante líquido.	0,80	1,00	1,00
Evite massas e doces.	0,80	0,80	0,73^*^
Busque rede de apoio com profissionais habilitados (psicólogo, fisioterapeuta, nutricionista, assistente social, enfermeiro, por exemplo).	0,93	1,00	1,00
Converse com familiares sobre medos, anseios, expectativas, sentimentos, preocupações.	1,00	0,93	0,87
Realize atividade física leve como pequenas caminhadas	0,80	0,93	0,93
Tome banho diariamente e use sabonetes neutros na região operada (ph 5,4 a 5,6).	0,80	0,87	0,87
Mantenha a pele limpa e hidratada.	0,60^*^	0,80	0,80
Troque a roupa diariamente.	0,87	0,78	0,86
Durma na posição dorsal (barriga pra cima) evitando acotovelamento do dreno e pressão sobre a ferida operatória.	0,93	1,00	1,00
Deixe o ambiente silencioso e tranquilo.	0,61^*^	0,61^*^	0,61^*^
Realize pequenas refeições frequentes para reduzir sensação de estômago cheio.	0,85	0,92	1,00
Evite deitar logo após uma alimentação	0,92	0,85	0,85
Faça uso da medicação caso tenha sido prescrita pelo médico.	0,71^*^	0,93	0,93
Identifique efeitos colaterais, posologia, horários.	0,57^*^	0,78	0,86
Leia a bula dos medicamentos prescritos.	0,46^*^	0,53^*^	0,53^*^
Observe aparecimento de sinais de alergia após uso dessas medicações e comunique a equipe. IVC dos itens sem IVC <0,78 IVC global sem IVC <0,78	0,77^*^ 0,86 0,90	0,85 0,90	0,85 0,91

CL - clareza de linguagem; PP - pertinência prática; RT - relevância teórica; IVC - Índice de Validade de Conteúdo; (^*^) IVC inferior ao ponto de corte (0,78).

Concluída essa etapa de avaliação por parte dos especialistas, modificações nos itens do guia seguiram as sugestões no tocante a alterações de algumas palavras por sinônimos apropriados, posição das palavras na frase, pontuação, ordem das informações, acréscimo de palavras para melhor explicação do item, mas também houve sugestões de agrupamento de itens semelhantes. Assim, a configuração final constou de 30 itens (Quadro 1).

**Quadro 1:** Itens do instrumento com orientações sobre autocuidado pós-operatório de cirurgia por câncer de mama após modificação, conforme sugestão dos especialistas, Fortaleza, Ceará, Brasil, 2022

Lave as mãos com água e sabão antes de tocar na ferida operatória.
Observe a ferida operatória para identificar possíveis sinais de infecção como vermelhidão, calor, secreção amarelada ou amarelo-esverdeada, febre.
Retire o curativo e deixe a ferida operatória descoberta após 24 horas da cirurgia.
Evite jato forte de água na pele e qualquer outro tipo de pressão sobre o local.
Mantenha a ferida operatória sempre limpa e seca evitando quaisquer produtos (óleos, pomadas, spray) a menos que orientada pela equipe de saúde.
Evite uso de fitas adesivas (esparadrapo, micropore) direto na pele ao redor do local da cirurgia.
Evite pegar sol na região da cirurgia. Tome banho todo dia e use sabonete líquido neutro (ph 5,4 a 5,6) na região operada.
Realize esvaziamento do dreno, medindo o volume da secreção retirada com uma seringa e anote no formulário fornecido na alta hospitalar. Logo após, despreze a secreção no sanitário.
Mantenha o dreno abaixo do local da cirurgia, evitando que fique direto no chão, pendurado ou dobrado.
Use roupas confortáveis, largas e abertas na frente para acomodar melhor o posicionamento do dreno. Troque-as diariamente.
Seque a pele ao redor do local onde o dreno está inserido, após o banho, com uma toalha limpa.
Durma com a barriga para cima evitando dobrar, acotovelar ou pressionar o dreno sobre a ferida operatória.
Evite exposição do braço do lado operado ao calor, machucados, queimaduras, aferição de pressão arterial e/ou glicemia capilar e/ou injeções e/ou retirada de sangue.
Evite retirada de cutículas das unhas das mãos do lado operado.
Use luvas de proteção térmica ao mexer no forno e luvas grossas ao mexer em plantas.
Use dedal ao costurar e meias elásticas no braço do lado operado conforme orientação profissional.
Evite pegar peso e não realize movimentos bruscos, de longa duração e repetitivo no braço do lado operado.
Diminua o consumo de gordura de origem animal na alimentação e mantenha as orientações nutricionais.
Dê preferência ao consumo de frutas, vegetais, hortaliças e mantenha as orientações nutricionais.
Beba bastante água, sucos naturais de frutas, evitando refrigerantes, bebidas alcoólicas e sucos artificiais.
Evite massas e doces e mantenha as orientações nutricionais.
Realize pequenas refeições ao longo do dia para reduzir sensação de estômago cheio.
Evite deitar logo após uma alimentação.
Busque rede de apoio com profissionais habilitados (psicólogo, fisioterapeuta, nutricionista, assistente social, enfermeiro, por exemplo).
Converse com familiares sobre medos, anseios, expectativas, sentimentos, preocupações.
Inicie fisioterapia o mais precoce possível e após liberação médica.
Realize atividade física leve como pequenas caminhadas, pilates, hidroginástica, ioga, preferencialmente após orientação de profissional habilitado.
Tome a medicação prescrita pelo médico.
Identifique possíveis efeitos colaterais e sinais de alergia das medicações tomadas, informando à equipe de saúde caso ocorra.

## DISCUSSÃO

A validação do guia de cunho educativo outrora apresentado demonstrou características importantes e significativas, sendo considerado adequado, ao passo que, na maioria dos itens, foi sugerido apenas ajuste na linguagem, que se refere exclusivamente quanto à forma de escrita da frase com foco na objetividade e clareza, sem uso de termos ambíguos, o que pode representar entendimentos inadequados e imprecisos^(29)^.

Nos outros dois domínios, PP e RT, ínfimas considerações foram pontuadas pelos especialistas, levando a duas conclusões: a primeira diz respeito à importância prática dos assuntos explorados no material, levando em conta que, conhecendo as particularidades da população-alvo e o problema atual de saúde, há empoderamento sobre o assunto abordado; e a segunda se refere à essencialidade da permanência dos itens no instrumento, visto cumprirem o propósito educativo eficiente a que se propôs.

Outra observação se refere à forma de medição da validade do conteúdo, pois, sendo realizada pelo IVC, permite analisar cada domínio, componente, item e o instrumento como um todo, considerando essa uma medida amplamente divulgada e aceita na literatura^(30)^.

A inclusão de outra categoria profissional da saúde como especialista se pautou nos princípios da interdisciplinaridade e multidisciplinaridade, além de que essa diversidade qualificou o percurso metodológico de uma validação de construto, tendo em vista que essa decisão contribuiu para a mitigação de resultados imprecisos, conclusões equivocadas ou medidas tendenciosas^(29)^.

Mesmo assim, enfermeiros compuseram uma parcela maior de avaliadores, certificando a enfermagem como ciência em construção pelo seu caráter de criação e mudança ao aliar conhecimentos científicos a procedimentos técnicos, e essa busca constante pela ampliação das esferas de cuidado traz perspectivas positivas para a profissão, a partir da intencionalidade do desenvolvimento de produtos capazes de proporcionar promoção, manutenção e reabilitação da saúde em cenários diversos.

Diante do processo educativo, o reconhecimento do indivíduo deve ser percebido como fator necessário às práticas de saúde, reorientando o modelo vigente e tornando-o partícipe da proposta de cuidado^(31)^.

No cenário oncológico mamário, desde o rastreamento e detecção precoce, estendendo-se por todo o processo de tratamento, o repasse de informações deve ocorrer de forma simples, já que comportamentos adequados de saúde podem ser conseguidos a partir do incentivo ao autocuidado, para, que dessa forma, haja possibilidade de autonomia do indivíduo^(32)^.

Nesse sentido, o paciente com câncer necessita de cuidados em que a condição de saúde é monitorada, controlada e pode ser acompanhada continuamente dentro de um processo educativo em busca do autocuidado e transformação de comportamento individual para promoção da saúde, sendo que essa medida é capaz de produzir mudanças significativas na capacidade de ajustamento à nova condição de saúde, inclusive com empoderamento pessoal^(33)^.

Entende-se que os níveis de autocuidado e gerenciamento dos pacientes oncológicos merecem ser aprimorados a partir do planejamento de enfermagem, a fim de melhorar os prognósticos sob o ponto de vista físico e aumentar sua flexibilidade psicológica para formar uma estrutura complementar benigna bidirecional de fisiologia e psicologia, já que a reabilitação inclui a integralidade do ser^(34)^. Dessa forma, muitas ferramentas podem ser utilizadas para o alcance da educação e promoção da saúde, mas, para isso, instrumentos educativos necessitam ser atualizados, ampliados, aprimorados e avaliados constantemente, considerando as fragilidades do momento.

### Limitações do estudo

Este estudo realizou apenas a validação do conteúdo de um guia de orientação, sendo excluídas outras formas de validação. Aponta-se, ainda, como limitação, o fato de os juízes especialistas serem de apenas duas categorias profissionais, enfermagem e fisioterapia, uma vez que a participação de outras categorias poderia trazer outros olhares, qualificando e ampliando a abrangência do material educativo.

### Contribuições para a área da enfermagem, saúde, ou política pública

As potenciais contribuições para o campo de práticas em saúde se assentam na possibilidade de o guia de orientação oferecer subsídios aos profissionais para a educação de pacientes no pós-operatório de câncer de mama, contribuindo para uma melhor recuperação e reabilitação dessas mulheres. Além disso, a sua utilização proporciona a disseminação de conhecimentos, podendo ser uma estratégia que fomente a realização do autocuidado.

## CONCLUSÕES

O conteúdo do guia de orientação sobre autocuidado pós-operatório no tratamento do câncer de mama foi considerado adequado e válido pelos especialistas. As sugestões realizadas pelos avaliadores foram incluídas e refinadas, estando aptas para aplicação na prática clínica junto à população de interesse.
